# The Active Ingredients and Potential Mechanism of Qijia Rougan Decoction in Autophagy and Hepatic Stellate Cell Activation Modulation in Liver Fibrogenesis

**DOI:** 10.1155/jamc/4646858

**Published:** 2025-05-12

**Authors:** Gui-Yu Li, Bai-Xue Li, Hong-Fei Song, Jie-Wen Gou, Li Wen, Quan-Sheng Feng

**Affiliations:** ^1^School of Basic Medical Sciences, Chengdu University of Traditional Chinese Medicine, Chengdu 610075, China; ^2^Department of Traditional Chinese Medicine, The Eighth Affiliated Hospital, Sun Yat-Sen University, Shenzhen 518033, China

**Keywords:** autophagy, hepatic stellate cells, liver fibrosis, Qijia Rougan decoction, traditional Chinese medicine

## Abstract

**Background and Objectives:** Liver fibrosis results from chronic inflammation. Qijia Rougan decoction, a traditional Chinese medicinal formulation, shows hepatoprotective potential, yet its mechanisms remain unclear. This study aims to investigate its antifibrotic effects and underlying mechanisms.

**Methods:** Rat liver fibrosis was induced by carbon tetrachloride (CCl_4_) and ethanol exposure. Histopathological assessment was performed using hematoxylin–eosin (HE) and Masson's trichrome staining. Hepatic stellate cell (HSC) activation and autophagic processes were examined through western blot analysis, immunofluorescence staining, and other in vitro assays. Components of Qijia Rougan decoction were analyzed by BATMAN-TCM platform. The pharmacological network was constructed using BATMAN-TCM platform, while disease-related targets were identified through DisGeNET database. Pathway enrichment analysis was conducted using KEGG pathway database.

**Results:** Significant reductions in hepatic index and serum biomarkers (ALT, AST, ALP, TBA, and γ-GT) were observed following Qijia Rougan decoction treatment, with maximal efficacy at 6 weeks. The decoction downregulated of LC3B and α-SMA expression in fibrotic tissues. In vitro, it suppressed LPS-induced α-SMA expression and autophagosome formation in HSC-T6 cells. Network pharmacology analysis of Qijia Rougan decoction identified 274 bioactive compounds and 12,883 potential targets, with pathway analysis indicating PI3K/AKT signaling as the predominant regulatory mechanism.

**Conclusion:** Qijia Rougan decoction alleviates liver fibrosis, potentially by inhibiting HSC activation and autophagy processes via PI3K/AKT/mTOR pathway.

## 1. Introduction

Liver fibrosis is the common pathological procession of chronic liver diseases, which, if left unchecked, can advance to cirrhosis and hepatocellular carcinoma. Etiologies of liver fibrosis encompass chronic hepatitis B, alcoholic liver diseases, and nonalcoholic fatty liver diseases, among others. The prevalence of alcohol-associated hepatitis and subsequent liver fibrosis appears to be on the rise globally, attributable to increased alcohol intake [1, 2]. Consequently, diseases related to liver fibrosis are emerging as a significant threat to public health [1].

The etiology of liver fibrosis is multifactorial, involving a complex interplay and cross-regulation among various cytokines, molecular signals, and signaling pathways. This complexity renders the development of therapeutics for liver fibrosis particularly challenging. Despite promising results in preclinical models, the efficacy of many antifibrotic agents in clinical settings remains equivocal. To date, no specific, potent, and safe medications have been approved for the clinical treatment of liver fibrosis [3, 4]. There is, therefore, an unmet need for more effective and safer antifibrotic therapies to enhance patient outcomes.

Qijia Rougan decoction, formulated by the renowned Chinese traditional medicine physician Professor Zhiwen Zhang, specializes in the treatment of chronic liver diseases. Qijia Rougan decoction is a traditional Chinese medicinal formulation comprising eight medicinal components: *Astragalus mongholicus* Bunge (Chinese name: Huangqi), *Trionyx sinensis* Wiegmann (Chinese name: Biejia), *Prunus persica* (L.) Batsch (Chinese name: Taoren), *Carthamus tinctorius* L. (Chinese name: Honghua), *Angelica sinensis* (Oliv.) Diels (Chinese name: Danggui), *Sparganium stoloniferum* (Buch.-Ham. ex Graebn.) Buch.-Ham. ex Juz. (Chinese name: Sanleng), *Curcuma phaeocaulis* Valeton (Chinese name: Ezhu), and *Glycyrrhiza uralensis* Fisch. ex DC. (Chinese name: Gancao). Previous studies have shown that the first seven of the aforementioned eight traditional Chinese medicines each possess antiliver fibrosis effects [5–10]. Based on Professor Zhang's extensive clinical expertise, the decoction is formulated to tonify healthy qi, promote blood circulation, and resolve blood stasis, demonstrating promising therapeutic potential for hepatoprotection and the amelioration of cirrhosis and fibrosis. However, the clinical application of Qijia Rougan decoction and the advancement of effective antiliver fibrosis drugs are impeded by a lack of clarity regarding its impact on liver fibrosis. Further investigation into the antifibrotic effects and mechanisms of Qijia Rougan decoction is thus warranted.

Clinical observations indicate that liver fibrosis is a dynamic and reversible process with the potential for recovery and remodeling. Central to the pathogenesis of hepatic fibrosis is the repetitive injury to hepatocytes, the activation of inflammatory responses postinjury, and the activation and proliferation of HSCs. HSC activation is a pivotal event in fibrosis development [11]. Autophagy has been shown to modulate HSC activation and proliferation, with interventions at various autophagic stages capable of inhibiting these processes [12, 13]. This study aims to explore the antiliver fibrosis effects, its potential active ingredients, and mechanisms of Qijia Rougan decoction through the establishment of a rat liver fibrosis model in vivo, an activated HSC model in vitro, and network pharmacology analysis.

## 2. Materials and Methods

### 2.1. Drugs and Reagents

Carbon tetrachloride was sourced from Tianjin Bodi Chemical Co., Ltd. (Tianjin, China). Peanut oil was obtained from Sichuan Kerry Grain and Oil Industry Co., Ltd. (Deyang, China). Paraformaldehyde and HRP-labeled sheep and rabbit antibodies were procured from Boster Biological Technology Co., Ltd. (Wuhan, China). Sodium pentobarbital and LPS were purchased in Sigma-Aldrich. Aspartate aminotransferase (AST) assay kit (IFCC method), alkaline phosphatase (ALP) assay kit (AMP buffer method), alanine aminotransferase (ALT) assay kit (IFCC method), total bile acid (TBA) assay kit (enzymatic cycling method) and γ-glutamyltransferase (γ-GT) assay kit (IFCC method) were acquired from Shenzhen Mindray Bio-Medical Electronics Co., Ltd. (Shenzhen China). Anhydrous ethanol was supplied by Chengdu Haixing Chemical Reagent Factory (Chengdu, China). Xylene and isopropanol were provided by Tianjin Zhiyuan Chemical Reagent Co., Ltd. (Tianjin, China). Hematoxylin and eosin (HE) dyes solution was purchased from Wuhan Servicebio Technology Co., Ltd. (Wuhan, China) and Baso Diagnostics Inc. Zhuhai (Zhuhai, China), respectively. Neutral gum was obtained from Labgic Technology Co., Ltd. (Anhui, China). The modified Masson Tri-color dyeing liquid was from Hefei Bomei Biotechnology Co., Ltd. (Hefei, China). Phosphatase inhibitor, RIPA lysate, and BCA protein concentration assay kits were purchased from Beyotime Biotech. Inc. (Shanghai, China). Phenylmethylsulfonyl fluoride (PMSF) was from Shanghai Aladdin Biochemical Technology Co., Ltd. (Shanghai, China). TEMED was sourced from Sinopharm Chemical Reagent Co., Ltd. (Shanghai, China). Rabbit polyclonal anti-α-smooth muscle actin (α-SMA), anti-alpha SMA, and recombinant anti–microtubule-associated protein 1A/1B-light chain 3B (LC3B) antibodies were purchased from Abcam (Cambridge, UK). Rabbit polyclonal antibody LC3 (14/16KD) was purchased from Proteintech Group, Inc. (Wuhan, China). ECL substrate solution was from Applygen Technologies Inc. (Beijing, China). Fetal bovine serum was acquired from Invitrogen/Gibco (New York, America). The antifluorescence quenching sealing solution was purchased from Beijing Solarbio Science & Technology Co., Ltd. (Beijing, China). The CCK8 kit was from DOJINDO (Kyushu Island, Japan). Cell culture media and reagents, including DMEM high-glucose medium, penicillin–streptomycin mixture, trypsin solution, and PBS buffer, were supplied from HyClone (Washington, D.C., USA). Beta-actin antibody and Hoechst 33258 dye solution were purchased from Shanghai Qiming Biotechnology Co., Ltd. (Shanghai, China) and Suzhou Yuheng Biotechnology Co., Ltd. (Suzhou, China), respectively. The 812 epoxy resin embedding kit, uranyl acetate, and lead citrate dye solution were from Beijing Zhongjingkeyi Technology Co., Ltd. (Beijing, China).

### 2.2. Composition and Preparation of Qijia Rougan Decoction

Qijia Rougan decoction is composed of the following components: *Astragalus mongholicus* Bunge (Huangqi), *Trionyx sinensis* Wiegmann (Biejia), *Prunus persica* (L.) Batsch (Taoren), *Carthamus tinctorius* L. (Honghua), *Angelica sinensis* (Oliv.) Diels (Danggui), *Sparganium stoloniferum* (Buch.-Ham. ex Graebn.) Buch.-Ham. ex Juz. (Sanleng), *Curcuma phaeocaulis* Valeton (Ezhu), and *Glycyrrhiza uralensis* Fisch. ex DC. (Gancao), in the ratio of 10:5:6:3:5:5:17, respectively. The herbs were sourced from Sichuan Xinglin Pharmaceutical Chain Co., Ltd. (Sichuan, China), soaked in tenfold double-distilled water for 30 min, and then decocted. *Trionyx sinensis* Wiegmann was decocted first. It was heated on high flame until boiling and then simmered on low heat for 1 h. Subsequently, the remaining herbs were added and the decoction continued for an additional 30 min. The preparation of lyophilized powder involved standardized extraction and purification protocols. After the initial decoction, the liquid extraction was concentrated with a rotary evaporator (RE-5210A, Shanghai Yarong Biochemical Instrument Factory, Shanghai, China) at 60°C under reduced pressure to obtain a concentrated solution with a concentration of 1.10–1.15 g/mL (measured at 25°C). The concentrated extract was filtered through three stages: Coarse particles were filtered through a 200-mesh nylon cloth, and fine impurities and microorganisms were filtered through 0.45-μm and 0.22-μm micropore membranes (Millipore, USA). The filtrate was then frozen in an ultra-low-temperature freezer (MDF-682, Panasonic, Japan) at −80°C for 12 h and then freeze-dried. Freeze-drying was performed using a freeze-dryer (FDU-2110, Tokyo Rikakikai, Japan) under the following conditions: primary drying at −50°C for 24 h under vacuum (0.1 mbar), followed by secondary drying at 25°C for 6 h. The freeze-dried powder was kept in an airtight container with a desiccant at 4°C until used. The entire process was conducted in a clean room (class 10,000) under sterile conditions to ensure product quality and stability. The decoction was then standardized into low, medium, and high dosages based on pre-experimental outcomes [14]. Our pre-experimental evaluation employed three doses of Qijia Rougan decoction concentrate: 1.2, 0.6, and 0.3 mL/100 g body weight. Since freeze-drying equipment was not available at that time, we administered the decoction in the form of concentrated liquid, in which 1 mL of concentrated liquid contained 2 g of crude drug. Following a 6-week period of intragastric administration, we found that all dose groups exhibited significant ameliorative effects on liver fibrosis, of which 1.2 mL/100 g dose group had the best effect without inducing any observable adverse effects (data not shown). It should be noted that the administered concentration of 1.2 mL/100 g, equivalent to 2.4 g crude drug/100 g (calculated as 1.2 mL/100 g × 2 g crude drug/mL), represents the most effective dosage in our experimental protocol. This result provides an important basis for dosage selection in formal experiments. In the formal experiment, we optimized the extraction process and used freeze-drying equipment. Based on the pre-experimental results, we converted the dose to the form of lyophilized powder: adjusted to high, medium, and low doses of 2.26, 1.13, and 0.57 g/100 g after optimizing the extraction process. These doses have taken into account the conversion from concentrated liquid to lyophilized powder, reducing the dose by about 5.8%. The reasons for adjustment are as follows: The new process improves the extraction efficiency of the active ingredient, the lyophilization process enhances the stability of the ingredient, and the dose optimization based on the pre-experimental results ensures safety and efficacy.

### 2.3. Animals and Experimental Protocol

All animal procedures were approved by the Institutional Animal Ethics Committee of Chengdu University of Traditional Chinese Medicine (Sichuan, China; approval #2021-66) and were conducted in strict accordance with the guidelines.

Sprague Dawley (SD) rats, weighing 150–180 g and aged two months, were sourced from Chengdu Dossy Experimental Animals Co., Ltd. (Chengdu, China). After a 1-week acclimation period, the rats were subjected to a regimen to induce liver fibrosis using CCl_4_ in a mixture with peanut oil and 10% alcohol for 8 weeks. The vehicle control group, consisting of 10 rats, was administered peanut oil subcutaneously and provided with pure water ad libitum. Additional 40 rats were subjected to a regimen to induce liver fibrosis via subcutaneous injections of a carbon tetrachloride (CCl_4_) and peanut oil mixture in a volume ratio of 2:3 for the initial 3 weeks, followed by a 1:1 ratio for the subsequent 5 weeks. The injection volume was 0.3 mL per 100 g of body weight, administered twice weekly. These rats were also given 10% alcohol to replace drinking water for a period of 8 weeks.

Upon successful induction of liver fibrosis, the 40 rats were randomly assigned to four groups: Groups 1 to 4. Group 1 rats were treated with normal saline via intragastric administration once daily for 6 weeks. Groups 2 to 4 received intragastric administrations of Qijia Rougan decoction at dosages of 0.57 g/100 g, 1.13 g/100 g, and 2.26 g/100 g, respectively, once daily for 6 weeks. Additionally, to prepare drug-containing serum, rats were treated with normal saline and three dosages of Qijia Rougan decoction (0.57 g/100 g, 1.13 g/100 g, and 2.26 g/100 g) for seven days.

Following the treatment period, the animals were anesthetized using sodium pentobarbital and euthanized in a humane manner. Blood samples were collected and centrifuged to obtain serum, and liver tissues were excised. Both serum and liver tissue samples were stored at −80°C for further analysis.

### 2.4. Liver Index and Serum Hepatic Enzyme Level Assessments

The liver index (= wet liver weight/body weight ∗ 100%) was recorded and calculated.

An automatic biochemical analyzer (BS-240VET, Shenzhen Mindray Bio-Medical Electronics Co., Ltd.) was utilized to measure serum levels of liver enzymes, including ALT, AST, ALP, TBA, and γ-GT content.

### 2.5. Histology Examination

The liver tissues were fixed in 4% paraformaldehyde for 24 h, processed through a graded ethanol series, cleared with xylene, embedded in paraffin, and sectioned at 3 μm thickness. Sections were stained with HE or Masson and examined under a light microscope. Collagen content was quantified using ImageJ software (the United States of America).

### 2.6. Immunofluorescent Analysis

Paraffin-embedded liver tissue sections were dewaxed, rehydrated, and subjected to antigen retrieval. Antigen repair: 0.01 mol/L sodium citrate was added into the repair box, heated for 10 min after high pressure, and cooled until room temperature. Removing endogenous enzymes: The slides were placed in a slide wet box, added H_2_O_2_, placed at room temperature for 10 min, and rinsed with PBS for 3 times (5 min/time). Closure: 10% normal donkey serum was added overnight at 4°C. Adding primary antibody: Dilute LC3B or α-SMA primary antibody was added (diluted at 1:200), incubated at 4°C overnight, rewarmed for 45 min the next day, and rinsed with PBS 3 times (5 min/time). Adding secondary antibodies: Dilute immunofluorescently labeled secondary antibodies were added, placed at room temperature for 1 h, and rinsed with PBS for 3 times (5 min/time). The Hoechst was added to the samples for nuclear staining at room temperature for 3 min. The dye solution was discarded, and the slices were washed with PBS 3 times. The samples were sealed with antifluorescence quenching tablets. Slides were examined under a confocal microscope (LEICA, TCS SP8 SR, Germany).

### 2.7. Western Blotting

Protein extraction and quantification were performed using RIPA buffer and BCA protein assay kit, respectively. Equal amounts of protein were separated by SDS-PAGE and transferred onto PVDF membranes. After incubation with primary antibodies against α-SMA and subsequent HRP-conjugated secondary antibodies, protein bands were visualized using an ECL substrate solution, and band intensities were analyzed using ImageJ.

### 2.8. Cells and Experimental Protocol

HSC-T6 cell lines were provided by BeNa Culture Collection Biotechnology Co., Ltd. (Henan, China) and cultured in DMEM high-glucose medium supplemented with 10% fetal bovine serum and 1% double antibody in a 5% CO_2_ incubator at 37°C. Cells were treated with varying concentrations of drug-containing serum derived from rats treated with Qijia Rougan decoction. According to the results of CCK8 experiment, 10% serum was selected as the serum concentration for the following treatment. HSC-T6 cell lines were divided into Group 1, Group 2, Group 3, Group 4, and Group 5. Cells in Group 1 (vehicle control group) received 10% blank rat serum. Cells in Group 2 (the model group) were incubated with LPS 0.1 mg/L [15] for 24 h. Cells in Group 3 (LPS + low-dosage drug-containing serum group) were incubated with LPS 0.1 mg/L for 24 h and treated with 10% low-dosage drug-containing serum for 24 h. Cells in Group 4 (LPS + medium-dosage drug-containing serum group) were incubated with LPS 0.1 mg/L for 24 h and treated with 10% medium-dosage drug-containing serum for 24 h. Cells in Group 5 (LPS + high-dosage drug-containing serum group) were incubated with LPS 0.1 mg/L for 24 h and treated with 10% high-dosage drug-containing serum for 24 h.

### 2.9. Cell Counting Kit-8 (CCK8)

HSC-T6 cells were seeded in 96-well plates, and the optimal serum concentration was determined using the CCK8 assay. After the cell growth reached about 70%, DMEM medium of 2.5%, 5%, 10%, 15%, and 20% blank rat serum (as experimental holes) was added into the 96-well plates of inoculated HSC-T6 cell lines, respectively. Control holes (Cell media without blank rat serum) and blank holes (cell-free and blank rat serum) were set up as experimental controls and treated for 24 h. Ten μL of CCK8 solution was added to each hole. After 2-h incubation in the incubator, OD values were detected at 450 nm to determine the appropriate serum concentration of rats. Cell survival rate = ([experimental hole OD value − blank hole OD value]/[control hole OD value − blank hole OD value]) ∗ 100%.

### 2.10. Transmission Electron Microscope

Cells were sectioned into 1-mm^3^ slices and incubated at 2.5% glutaraldehyde at 4°C for 12 h. After washing with PBS, the samples were fixed with 1% osmic acid at 4°C for 3 h. The 50-nm slices were dehydrated with ethanol and acetone, then buried in epoxy resin for 4 h, and stained with uranium acetate and lead citrate. Autophagosomes were observed under a transmission electron microscope (JEOL JEM-1400 plus, Japan).

### 2.11. Network Pharmacology Analysis

Targets associated with liver fibrosis were predicted using the DisGeNET platform (https://www.disgenet.org), which integrates diverse datasets from authoritative genomic sources, GWAS data, animal models, and the scientific literature to identify genes linked to human pathologies.

The BATMAN-TCM tool (https://bionet.ncpsb.org/batman-tcm/) was employed to predict the molecular targets of Qijia Rougan decoction. This online bioinformatics platform is designed to analyze the molecular mechanisms of traditional Chinese medicinal formulas [16]. The pinyin names of the constituent herbs were inputted, with a cutoff value set to 10 to identify potential targets. Subsequently, the predicted targets were refined for accuracy using the UniProt database (https://www.uniprot.org/).

The intersection of targets for Qijia Rougan decoction and liver fibrosis was determined utilizing the Venn diagram tool (https://bioinformatics.psb.ugent.be/webtools/Venn/). The String platform (https://string-db.org/) was then used to map the protein–protein interaction (PPI) network, with parameters set for “*Homo sapiens,*” and a confidence score threshold of 0.9. Cytoscape 3.7.0 software was subsequently used to construct the Qijia Rougan decoction—liver fibrosis target interaction network from the PPI data.

Gene ontology (GO) functional enrichment analysis was performed on the core target genes using the ClusterProfiler package within the R 3.6.1 environment. GO annotations facilitate the classification and retrieval of genes and proteins based on their biological characteristics across three domains: molecular function, cellular component, and biological process (BP) [17, 18].

Enrichment analysis of the intersecting targets within the Kyoto Encyclopedia of Genes and Genomes (KEGG) pathways was conducted using the ClusterProfiler package in R 3.6.1. The analysis employed a hypergeometric distribution model for pathway enrichment and the Benjamini–Hochberg procedure for multiple test correction and significance assessment.

### 2.12. Statistical Analysis

Statistical analysis was performed using GraphPad Prism 5.0 software. Data are expressed as the mean ± standard deviation (SD). Intergroup differences were assessed using the one-way analysis of variance (ANOVA), complemented by a post hoc multiple comparison test. Statistical significance was set at *p* < 0.05, with *p* < 0.01 and *p* < 0.001 indicating high and very high significance, respectively.

## 3. Results

### 3.1. Qijia Rougan Decoction Decreased Levels of Liver Index and Serum Hepatic Enzyme Levels

**Figure 1:**
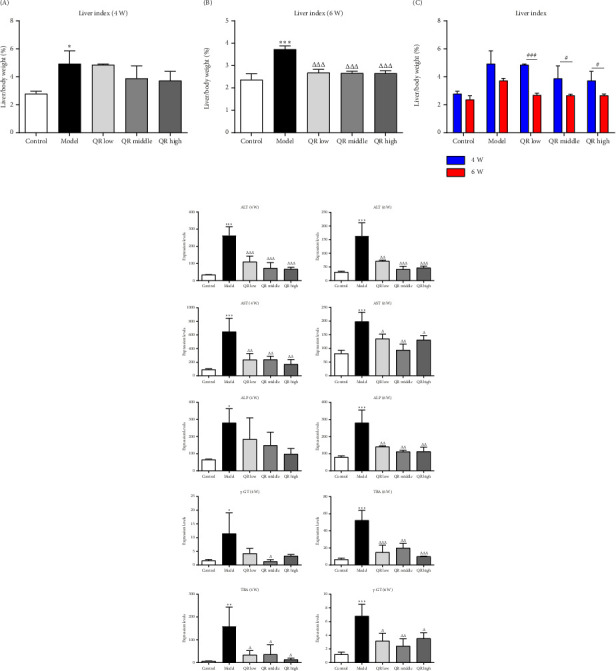
Qijia Rougan decoction reduced liver index and liver function index in rats with liver fibrosis. (a) Qijia Rougan decoction reduced liver index. (A) Results of 4-week treatment. (B) Results of 6-week treatment. (C) Comparison between four and 6 weeks of treatment. (b) Levels of ALT, AST, TBA, and γ-GT after 4 weeks of Qijia Rougan decoction treatment. (c) Levels of ALT, AST, TBA, and γ-GT after 6 weeks of Qijia Rougan decoction treatment. Statistical analysis was conducted using the one-way analysis of variance (ANOVA) with a post hoc test for multiple comparisons. *VS* vehicle, ^∗^*p* < 0.05, ^∗∗^*p* < 0.01, ^∗∗∗^*p* < 0.001. *VS* model group, ^△^*p* < 0.05, ^△△^*p* < 0.01, ^△△△^*p* < 0.001. ^#^*p* < 0.05, ^##^*p* < 0.01, ^###^*p* < 0.001. QR, Qijia Rougan decoction.

After 4 weeks of treatment, liver index and liver function indexes (ALT, AST, ALP, TBA, and γ-GT) in the model group increased compared with the control group (Figures 1(a)-A and 1(b)). The liver index exhibited a decreasing trend after treatment with Qijia Rougan decoction at low, medium, and high doses (Figures 1(a)-B and 1(a)-C), and however, these changes did not reach statistically significance.

Significant reductions were observed in the levels of ALT, AST, and TBA within the low dose of Qijia Rougan decoction (Figure 1(b)). Similar decreases were noted for ALT, AST, TBA, and γ-GT in the medium-dose decoction and for ALT, AST, and TBA in the high-dose decoction (Figure 1(b)). After a 6-week treatment duration, the model group demonstrated significantly higher levels of liver index, ALT, AST, ALP, TBA, and γ-GT compared to the control group (Figure 1(c)). Comparatively, all tested dosages of Qijia Rougan decoction led to a reduction in the liver index and the aforementioned liver function parameters, with the therapeutic effects being more pronounced after the 6-week treatment duration than after 4 weeks.

### 3.2. Qijia Rougan Decoction Improved CCl_4_− and Alcohol-Induced Liver Fibrosis in Rats

**Figure 2:**
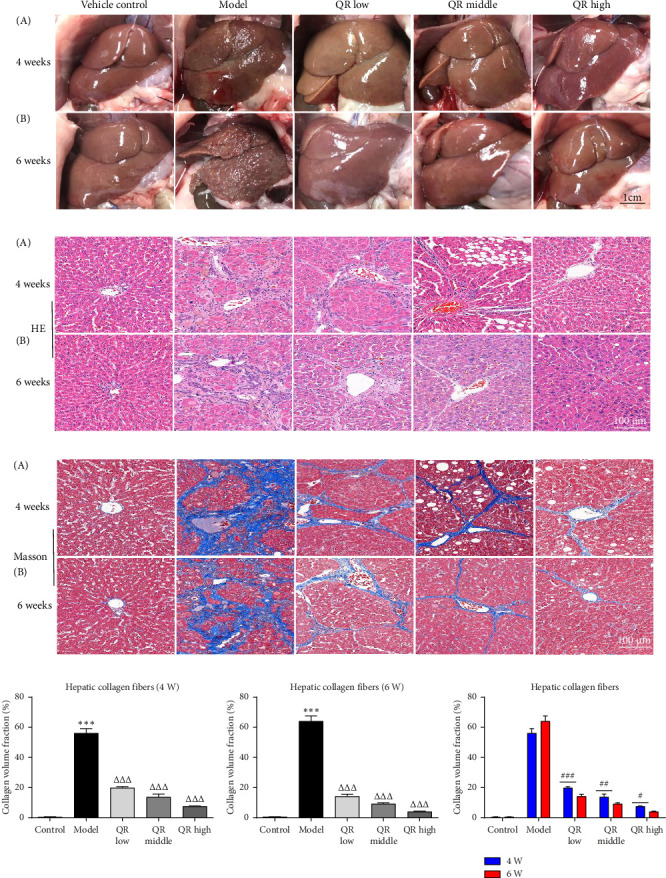
Qijia Rougan decoction ameliorated CCl_4_- and alcohol-induced liver fibrosis in rats. (a) Liver surface views of rats from each group after 4 weeks (A) and 6 weeks (B) of treatment. (b) HE staining of liver tissue from rats after 4 weeks (A) and 6 weeks (B) of treatment. (c) Masson's trichrome staining of liver tissue from rats. (d) Hepatic collagen fibers volume fraction based on the results from (c). Statistical significance was assessed using one-way ANOVA with a post hoc multiple comparison test. *VS* vehicle, ^∗∗∗^*p* < 0.001. *VS* model group, ^△△△^*p* < 0.001. ^#^*p* < 0.05, ^##^*p* < 0.01, ^###^*p* < 0.001. QR, Qijia Rougan decoction.

Macroscopic examination of the liver in the vehicle control group revealed a bright red color, smooth surface, well-defined edges, and a soft consistency (Figure 2(a)). In contrast, the model group livers, characterized by CCl_4_− and alcohol-induced fibrosis, appeared darker, with a miliary convex surface, and were dull-edged and firm in texture (Figure 2(a)). Post-treatment with Qijia Rougan decoction for four or 6 weeks, the hepatic tissues in the low-, medium-, and high-dose groups exhibited a reversal to a bright red color, smooth surface, well-defined edges, and soft texture when compared to the model group (Figure 2(a)). Histological analysis using Masson's trichrome and HE staining demonstrated excessive collagen deposition around the interlobular arteries, veins, and bile ducts in the model group, with thickened fibers forming bridges and encapsulating hepatic lobules, leading to the formation of “pseudolobules” (Figures 2(b) and 2(c)). Treatment with Qijia Rougan decoction at all dosages resulted in thinner collagen fibers and reduced collagen content in the liver tissue, with the high dosage showing the most significant effects (Figures 2(b) and 2(c)). The therapeutic outcomes were enhanced with the 6-week treatment duration compared to the 4-week period (Figure 2(d)).

### 3.3. Qijia Rougan Decoction Inhibited Both Activation of HSCs and Autophagy in Rats and in HSC-T6

To ascertain the optimal serum concentration for cell culturing, the CCK8 assay was conducted to assess cell viability with varying concentrations of blank rat serum. The findings revealed a marked decrease in cell viability at serum concentrations above 15% in comparison with the control group. Conversely, concentrations ranging from 0% to 10% did not exhibit a significant deviation in cell viability, suggesting that these levels do not negatively impact cellular vitality (data not shown). Consequently, a serum concentration of 10% was chosen for use in subsequent in vitro experiments.

**Figure 3:**
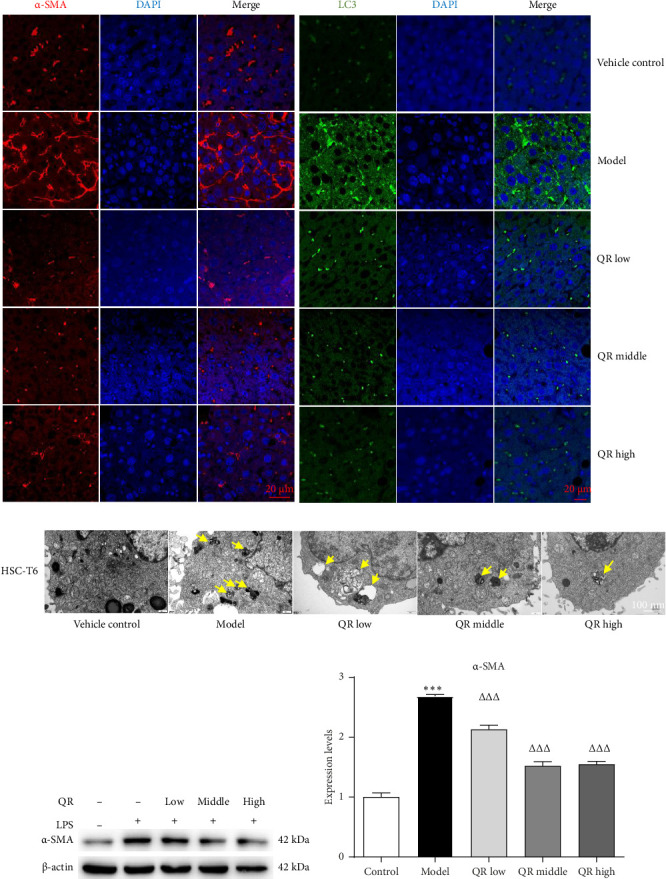
Qijia Rougan decoction inhibited activation of HSCs and autophagy in liver fibrosis in rats and HSC-T6 cells. (a) Immunofluorescence results in rats. α-SMA is shown in red, LC3 in green and nucleus in blue. (b) Transmission electron microscope results in HSC-T6 cells. (c) Quantitative analysis of α-SMA band intensity. (d) WB analysis of α-SMA protein expression in HSC-T6 cells. Bar graphs were constructed based on data from (c). Statistical significance was determined using one-way ANOVA with a post hoc test. *VS* vehicle, ^∗∗∗^*p* < 0.001. *VS* model group, ^△△△^*p* < 0.001. QR, Qijia Rougan decoction.

Immunofluorescence staining results indicated that the expression of α-SMA, a marker for HSC activation, and LC3B, a marker for autophagy activation, was significantly elevated in the liver tissue of rats with CCl_4_- and alcohol-induced liver fibrosis (Figure 3(a)). Treatment with Qijia Rougan decoction at various dosages suppressed the levels of both α-SMA and LC3B (Figure 3(a)). In vitro studies using the HSC-T6 cell line corroborated these effects, demonstrating the decoctions' ability to inhibit HSC activation and autophagy (Figures 3(b), 3(c), and 3(d)).

### 3.4. Liver Fibrosis Target Network Analysis

**Figure 4:**
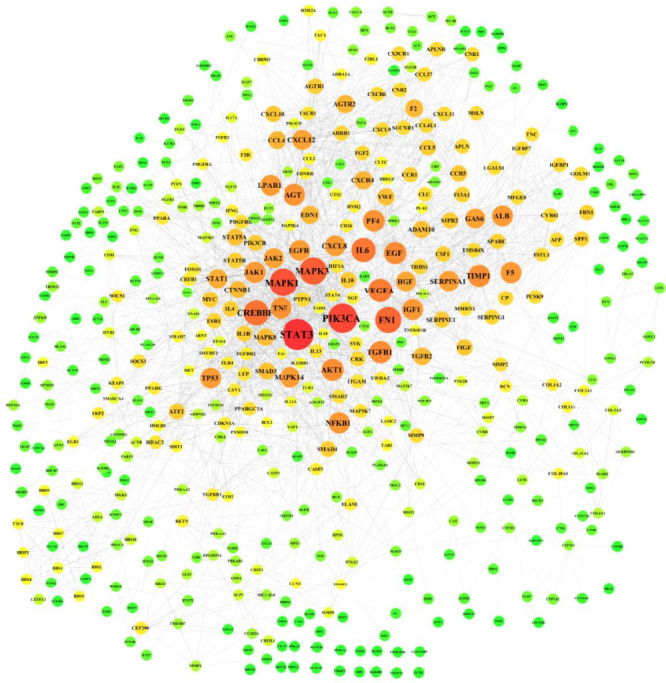
PPI network map of liver fibrosis. The size of nodes in the PPI network corresponds to the degree of connectivity, and larger nodes and more red color indicate a higher degree of interaction and more edges connected to the node.

A comprehensive analysis identified 1179 targets associated with liver fibrosis, with the PPI network comprising 1048 nodes and 27,524 edges (Figure 4). The top 10 genes with the highest degree of connectivity were STAT3 (74), PIK3CA (68), MAPK1 (65), MAPK3 (61), FN1 (58), CREBBP (55), IL6 (53), VEGFA (52), EGF (47), and TGFB1 (47).

### 3.5. Chemical Compounds of Qijia Rougan Decoction

Through the application of BATMAN-TCM, a database for traditional Chinese medicine, we identified a total of 274 compounds and 12,883 targets associated with Qijia Rougan decoction (Table 1). *Astragalus mongholicus* Bunge (AM) contained 34 compounds targeting 961 genes. *Trionyx sinensis* Wiegmann (CTC) was associated with 1 compound targeting six genes. *Carthamus tinctorius* L. (CTRR) had 30 compounds targeting 1140 genes. *Prunus persica* (L.) Batsch (PP) contained three compounds targeting 155 genes. *Angelica sinensis* (Oliv.) Diels (AS) was linked to 96 compounds targeting 7073 genes. *Sparganium stoloniferum* (Buch.-Ham. ex Graebn.) Buch.-Ham. ex Juz. (SS) had eight compounds targeting 568 genes. *Curcuma phaeocaulis* Valeton (RC) contained seven compounds targeting 844 genes. *Glycyrrhiza uralensis* Fisch. ex DC. (GU) was associated with 95 compounds targeting 2136 genes.

### 3.6. Intersection Target Network of Qijia Rougan Decoction and Liver Fibrosis

**Figure 5:**
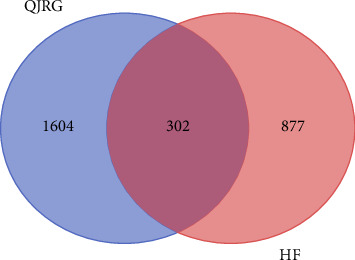
Intersection genes of Qijia Rougan decoction and liver fibrosis. QJRG, Qijia Rougan decoction; HF, hepatic fibrosis.

**Figure 6:**
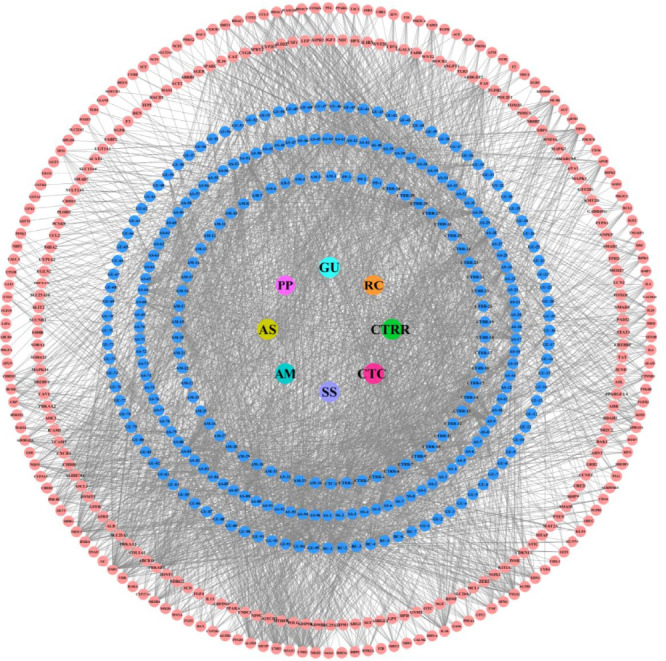
Intersection target network of Qijia Rougan decoction and liver fibrosis. The Qijia Rougan decoction's therapeutic target network in the context of liver fibrosis. The glue color signifies the intersection of the herbal compounds from the decoction that intersect with the disease, while red delineates the specific molecular targets implicated at this intersection. AM, *Astragalus mongholicus* Bunge; CTC, *Trionyx sinensis* Wiegmann; CTRR, *Carthamus tinctorius* L.; PP, *Prunus persica* (L.) Batsch; AS, *Angelica sinensis* (Oliv.) Diels; SS, *Sparganium stoloniferum* (Buch.-Ham. ex Graebn.) Buch.-Ham. ex Juz.; RC, *Curcuma phaeocaulis* Valeton; GU, *Glycyrrhiza uralensis* Fisch. ex DC.

Venn diagram intersection analysis revealed that 302 genes intersected between the eight constituent herbs of Qijia Rougan decoction and liver fibrosis targets (Figure 5). The intersection target network between Qijia Rougan decoction and liver fibrosis is depicted in Figure 6, with the following compounds intersecting with hepatic fibrosis targets: 31 out of 34 compounds in AM, 1 out of 1 compound in CTC, 27 out of 30 compounds in CTRR, 3 out of 3 compounds in PP, 88 out of 96 compounds in AS, all 8 compounds in SS, all 7 compounds in RC, and 90 out of 94 compounds in GU.

### 3.7. Enrichment Analysis of Intersection Target Network Between Qijia Rougan Decoction and Liver Fibrosis

**Figure 7:**
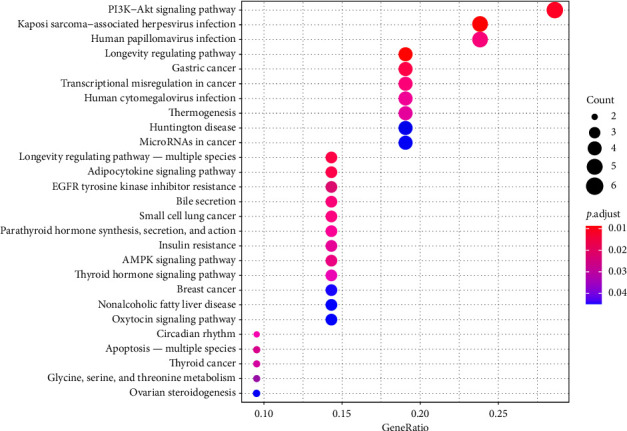
Bubble map of KEGG enrichment pathway at the intersection of Qijia Rougan decoction and liver fibrosis. The bubble map illustrates pathways significantly enriched in relation to key target genes. The size of the bubble represents the number of genes enriched in the pathway; larger bubbles indicate more genes are involved.

**Figure 8:**
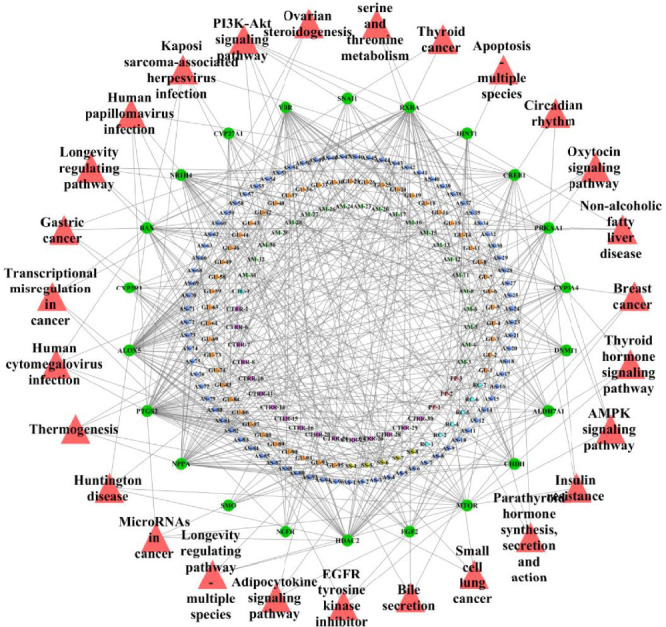
KEGG pathway–target–compound topology at the intersection of Qijia Rougan decoction and liver fibrosis. The red color indicates KEGG-enriched signaling pathways, green denotes the intersecting key genes, and the three innermost circles represent the herbal compounds of the decoction.

GO enrichment analysis, with a significance threshold of *p* = 0.05, identified 3641 functional sets within the intersection target of Qijia Rougan decoction and liver fibrosis (data not shown). The BPs included negative regulation of autophagy, macroautophagy, HSC activation, and KEGG pathway analysis; conducted using R3.6.1 with a significance threshold of *p* = 0.05; and associated with a total of 27 pathways with liver fibrosis (Figure 7). All herbs in Qijia Rougan decoction were implicated in the modulation of these pathways (Figure 8). Notably, the PI3K-AKT signaling pathway was the most significantly enriched, suggesting its pivotal role in the therapeutic effects of Qijia Rougan decoction on liver fibrosis.

## 4. Discussion

The present study elucidates the hepatoprotective effects of Qijia Rougan decoction, demonstrating its efficacy in reducing liver enzymes and alleviating liver fibrosis. Our findings, derived from animal and cellular models as well as network pharmacology, shed light on the potential mechanisms underlying the decoction's impact on HSC regulation and autophagy activation.

Histological assessments via HE and Masson's trichrome staining revealed that Qijia Rougan decoction effectively reversed hepatic fibrosis, significantly diminished collagen fiber deposition, and reduced the severity of fibrosis. Notably, the therapeutic effects were more pronounced after 6 weeks of treatment compared to 4 weeks. These observations suggest that Qijia Rougan decoction significantly ameliorates liver fibrosis, with a more pronounced effect observed over a longer treatment duration.

Serum enzyme levels, including ALT, AST, and ALP, are established biomarkers of liver function and injury [19]. Elevated levels of these enzymes in the serum are indicative of hepatocellular damage [20]. In the model group, significant increases in ALT, AST, and ALP were observed, which were attenuated with Qijia Rougan decoction treatment, especially at the high dosage (Figure 1(b)). After 4 weeks, AST and ALT levels were significantly decreased, while ALP showed a decreasing trend, albeit not statistically significant. By 6 weeks, a significant reduction in all three enzymes was evident, suggesting that Qijia Rougan decoction reduces hepatic enzyme activity and protects liver cells from damage. The specificity of ALT for liver injury makes it a more reliable indicator of hepatic damage than AST, despite its presence in other tissues [21], although the reduction in ALT levels did not significantly differ between the four- and 6-week treatments, possibly due to its high sensitivity to the presence of significant liver fibrosis rather than its specificity to the degree of fibrosis [22].

TBA is synthesized from cholesterol in the liver and plays a critical role in the excretion of hydrophobic compounds and the absorption of fats [23]. TBA levels are sensitive indicators of liver injury in chronic hepatitis, and high levels are associated with significant liver fibrosis [24]. Qijia Rougan decoction treatment led to a significant reduction in TBA levels, suggesting a protective effect on liver damage. γ-GT, synthesized and secreted by hepatocyte mitochondria, is another marker of liver injury [25]. Increased levels of γ-GT in the serum are indicative of liver fibrosis [26]. Qijia Rougan decoction treatment significantly reduced γ-GT levels, further supporting its protective role against liver damage.

The liver index, which is the ratio of liver wet weight to body mass, was significantly higher in the model group, indicating potential hyperemia, swelling, injury, or compensatory hyperplasia of the liver. Qijia Rougan decoction significantly reduced the liver index, suggesting its protective function against liver injury and its ability to alleviate liver injury.

HSC activation is a pivotal even in the pathogenesis of liver fibrosis [13]. In our CCl_4_-induced rat model of liver fibrosis, immunofluorescence staining revealed a substantial presence of α-SMA, an HSC activation marker, within the fibrotic liver tissue. This finding indicates active HSCs and the establishment of liver fibrosis. Treatment with Qijia Rougan decoction led to a significant reduction in α-SMA expression within fibrotic liver tissue, as assessed by immunofluorescence staining. Collectively, these observations suggest that Qijia Rougan decoction may inhibit HSC activation, thereby preventing the progression of liver fibrosis.

Autophagy, derived from the Greek meaning “self-eating,” is a cellular process of self-digestion, initially characterized by Christian de Duve approximately 4 decades ago following studies on rat liver lysozyme infused with glucagon [27]. The role of autophagy in liver diseases, including fibrosis, is complex and varies among studies. While some research highlights a protective role for autophagy in liver health, other work suggests it may contribute to cell death and disease progression [28]. Recent studies have underscored the significance of autophagy in regulating HSC activation and its influence on the development of liver fibrosis [13, 29–34]. Our study's results indicate that in rats with liver fibrosis, both autophagy marker LC3B and HSC activation marker α-SMA were highly expressed. Following Qijia Rougan decoction administration, a decrease in LC3B and α-SMA expression was observed, along with a reduction in liver fibrosis lesions. These results imply that Qijia Rougan decoction may exert its antifibrotic effects by downregulating HSC autophagy and inactivating HSCs.

Utilizing network pharmacology, we constructed a disease (liver fibrosis)–target–drug (Qijia Rougan decoction) interaction network. This analysis revealed numerous compounds and targets within Qijia Rougan decoction, with intersections observed between all eight constituent Chinese herbs (AM, CTC, CTRR, PP, AS, SS, RC, and GU) and liver fibrosis targets. Qijia Rougan decoction is hypothesized to modulate a range of BPs, including HSC activation, autophagy, fibroblast proliferation, inflammatory responses, and oxidative stress, potentially through interactions with the TOR complex and phosphatidylinositol-3-kinase (PI3K) activity, among others. Enrichment analysis of the KEGG pathway suggested that the PI3K/AKT signaling pathway may be a principal route through which Qijia Rougan decoction ameliorates liver fibrosis. The mammalian target of rapamycin (mTOR), a critical regulator of autophagy and a downstream effector of the PI3K/AKT pathway, is known to negatively regulate autophagy and plays a significant role in the pathogenesis of liver fibrosis [35–39]. In experimental models of hepatic fibrosis, downregulation of PI3K/AKT/mTOR signaling coincided with autophagy activation and HSC activation. Specifically, the PI3K inhibitor LY294002 was shown to inhibit PI3K and its downstream targets AKT and mTOR, thereby promoting autophagy and increasing the levels of autophagic lysosomes, LC3II, and Beclin1, as well as their mRNA expression [30]. Rapamycin, an autophagy agonist that primarily targets mTORC1, has been shown to activate autophagy and promote HSC activation in liver fibrosis [30, 40]. After treatment with rapamycin, a significant decrease in AKT and mTOR phosphorylation was observed, alongside increased autophagy-related protein expression, including ULK1 and ATG7, and elevated α-SMA [15, 41]. These findings indicate that mTOR pathway inhibition can activate HSC autophagy and upregulate the expression of HSC activation markers, suggesting that Qijia Rougan decoction may improve liver fibrosis by regulating HSC autophagy and activation through the PI3K/AKT/mTOR signaling pathway. Further research is warranted to delineate the specific mechanisms by which Qijia Rougan decoction inhibits HSC activation and autophagy in the context of liver fibrosis via the PI3K/AKT/mTOR pathway.

## 5. Conclusions

In summary, Qijia Rougan decoction was found to reduce the liver index, alleviate liver dysfunction, and mitigate liver fibrosis. The underlying mechanisms appear to involve the inhibition of HSC autophagy and activation, potentially through the PI3K/AKT/mTOR pathway, based on integrated in vivo, in vitro, and network pharmacology analyses. Future research is warranted to further delineate the specific mechanisms by which Qijia Rougan decoction modulates these pathways to combat liver fibrosis.

## Figures and Tables

**Table 1:** Compounds of Qijia Rougan decoction.

Herbs	Number of compounds	Number of target genes	Compounds
*Astragalus mongholicus* Bunge (AM)	34	961	Astragaloside VI (AM-1), 2-hydroxy-3-methoxystrychnine (AM-2), choline (AM-3), uridine (AM-4), astragaloside II (AM-5), chrysanthemaxanthin (AM-6), soyasaponin 1 (AM-7), lupeol (AM-8), cycloastragenol (AM-9), 20-hexadecanoylingenol (AM-10), guanosine (AM-11), formononetin (AM-12), isorhamnetin (AM-13), astramembrannin I (AM-14), beta-sitosterol (AM-15), astragaloside IV (AM-16), rhamnocitrin (AM-17), suffruticoside A (AM-18), betaine (AM-19), soyasapogenol B (AM-20), kaempferol (AM-21), quercetin (AM-22), kumatakenin (AM-23), astragaloside V (AM-24), astragaloside III (AM-25), gamma-sitosterol (AM-26), astragaloside I (AM-27), 3,5-dimethoxystilbene (AM-28), calycosin (AM-29), adenine (AM-30), sucrose (AM-31), acetic acid (AM-32), canavanine (AM-33), astragaloside VII (AM-34).
*Trionyx sinensis* Wiegmann (CTC)	1	6	Vitamin D (CTC-1)
*Carthamus tinctorius* L. (CTRR)	30	1140	Carvacrol (CTRR-1), carthamin (CTRR-2), dehydroshikimic acid (CTRR-3), 20-hexadecanoylingenol (CTRR-4), stearin (CTRR-5), 1-hexadecene (CTRR-6), alpha-onocerin (CTRR-7), 1-heptadecene (CTRR-8), carthamone (CTRR-9), linoleyl acetate (CTRR-10), safranal (CTRR-11), sagittariol (CTRR-12), hydroxysafflor yellow A (CTRR-13), neocembrene (CTRR-14), clerosterol (CTRR-15), 3-hexanol (CTRR-16), 3,4-dihydroxyrottlerin (CTRR-17), kaempferol (CTRR-18), quercetin (CTRR-19), 2-hexanol (CTRR-20), carthamidin (CTRR-21), chlorohydrin (CTRR-22), neocarthamin (CTRR-23), gamma-sitosterol (CTRR-24), phenylacetaldehyde (CTRR-25), onjixanthone II (CTRR-26), safflomin A (CTRR-27), arachidic acid (CTRR-28), lauric aldehyde (CTRR-29), onjixanthone I (CTRR-30).
*Prunus persica* (L.) Batsch (PP)	3	155	(+)-Catechin (PP-1), cordycepin (PP-2), amygdalin (PP-3).
*Angelica sinensis* (Oliv.) Diels (AS)	96	7073	Dimethyl phthalate (AS-1), beta-myrcene (AS-2), ethanol (AS-3), tetradecane (AS-4), carvacrol (AS-5), 2′,4′-dihydroxyacetophenone (AS-6), uridine (AS-7), beta-elemene (AS-8), azelaic acid (AS-9), chrysanthemaxanthin (AS-10), 2-methyl-dodecane-5-one (AS-11), 24,24-dimethyl-5alpha-cholesta-8-en-3beta-ol (AS-12), scopolin (AS-13), dimethyl azelate (AS-14), guanosine (AS-15), dodecenoic acid (AS-16), dimethyl-beta-propiothetin (AS-17), 4-ethylresorcinol (AS-18), vanillin (AS-19), dimethyl sebacate (AS-20), phenylacetic acid (AS-21), dictamnine (AS-22), 1-tridecene (AS-23), sebiferic acid (AS-24), alpha-terpineol (AS-25), 6-O-E-feruloylajugol (AS-26), scopoletin (AS-27), angelicin (AS-28), ethyl-P-methoxycinnamate (AS-29), decanoic acid (AS-30), 12-O-nicotinoylisolineolone (AS-31), dihydropinosylvin (AS-32), angelicide (AS-33), bicycloelemene (AS-34), alloocimene (AS-35), isoimperatorin (AS-36), 1-methyl-2-dodecyl-4-(1h)-quinolone (AS-37), limonene (AS-38), beta-bisabolene (AS-39), alpha-pinene (AS-40), naphthalene (AS-41), hexadecanoic acid (AS-42), 6-undecanol (AS-43), M-ethylphenol (AS-44), carvacrol acetate (AS-45), alpha-acoradiene (AS-46), adenine (AS-47), isococculidine (AS-48), O-cresol (AS-49), retinol (AS-50), 1,2-benzenedicarboxylic acid (AS-51), stigmasteryl ferulate (AS-52), eucalyptin (AS-53), dimethyl camphorate (AS-54), choline (AS-55), 3(S)-3-butyl-4,5-dihydrophthalide (AS-56), cedrol (AS-57), 1-tetradecanol (AS-58), anisic acid (AS-59), phyllanthin (AS-60), uracil (AS-61), pentylbenzene (AS-62), camphene (AS-63), 20-hexadecanoylingenol (AS-64), linalyl acetate (AS-65), P-cresol (AS-66), vitamin B1 (AS-67), 2-methyl-3-buten-2-ol (AS-68), 1,2-dimethylbenzene (AS-69), cnidilide (AS-70), beta-acoradiene (AS-71), butanoic acid (AS-72), decanal (AS-73), brefeldin A (AS-74), stigmasterol (AS-75), nonanal (AS-76), vitamin B12 (AS-77), cnidilin (AS-78), 1-hexadecanol (AS-79), 4-octanone (AS-80), P-ethylphenol (AS-81), campherenol (AS-82), beta-caryophyllene (AS-83), 1-dodecene (AS-84), guaiacol (AS-85), phenol (AS-86), isoeugenol (AS-87), M-cresol (AS-88), isofernene (AS-89), valerosidatum (AS-90), hexanoic acid (AS-91), crinamine (AS-92), 6-undecanone (AS-93), alpha-chamigrene (AS-94), butanal (AS-95), 3-carene (AS-96).
Sparganium stoloniferum (Buch.-Ham. ex Graebn.) Buch.-Ham. ex Juz. (SS)	8	568	Betulinic acid (SS-1), 5,7,2′,3′-tetrahydroxyflavone (SS-2), kaempferol (SS-3), sanshool (SS-4), betulin (SS-5), lupeol (SS-6), betulinaldehyde (SS-7), sanleng acid (SS-8).
*Curcuma phaeocaulis* Valeton (RC)	7	844	Neocurdione (RC-1), procurcumenol (RC-2), curcumin (RC-3), curdione (RC-4), curzerenone (RC-5), epicurzerenone (RC-6), beta-elemene (RC-7).
*Glycyrrhiza uralensis* Fisch. ex DC. (GU)	95	2136	18beta-glycyrrhetinic acid (GU-1), glycyrrhetinic acid (GU-2), ruvoside (GU-3), corylifolinin (GU-4), licoricesaponine F3 (GU-5), 18alpha-glycyrrhetinic acid (GU-6), methyl linoleate (GU-7), 4′-O-methylglabridin (GU-8), monoammonium glycyrrhizinate (GU-9), isoliensinine (GU-10), sinapic acid (GU-11), licoricesaponine C2 (GU-12), licorisoflavan A (GU-13), isoramanone (GU-14), gancaonin F (GU-15), glycyrol (GU-16), licoricesaponine A3 (GU-17), 3,3′-dimethylquercetin (GU-18), dimethyl sebacate (GU-19), neoisoliquiritin (GU-20), isotrilobine (GU-21), licoricesaponine J2 (GU-22), 2,5-dihydroxymethyl-3,4-dihydroxypyrrolidine (GU-23), gloeosteretriol (GU-24), narwedine (GU-25), tetrahydroharmine (GU-26), glycyrrhiza-flavonol A (GU-27), gancaonin A (GU-28), 2-methyl-1,3,6-trihydroxyanthraquinone (GU-29), glycyrin (GU-30), neohancoside A (GU-31), liquiritigenin-7,4′-diglucoside (GU-32), gancaonin I (GU-33), 8-methyl-10-hydroxylycoctonine (GU-34), methylglyoxal (GU-35), hispaglabridin A (GU-36), isoglycyrol (GU-37), lupiwighteone (GU-38), glyzaglabrin (GU-39), glycyrrhetol (GU-40), glycyrrhizic acid (GU-41), 2,4,4′-trihydroxychalcone (GU-42), 3′-methoxyglabridin (GU-43), alpha-trihydroxy coprostanic acid (GU-44), gancaonin P-3′-methylether (GU-45), licoricidin (GU-46), glisoflavanone (GU-47), licochalcone A (GU-48), hispaglabridin B (GU-49), isolicoflavonol (GU-50), licoricone (GU-51), phebalosin (GU-52), urea (GU-53), glyyunnanprosapogenin D (GU-54), 3-hydroxyglabrol (GU-55), licoflavone (GU-56), glycyrrhizin (GU-57), licobenzofuran (GU-58), formononetin (GU-59), licoricesaponine G2 (GU-60), isoliquiritin (GU-61), licoricesaponine D3 (GU-62), phaseollinisoflavan (GU-63), ferulic acid (GU-64), licoricesaponin C2 (GU-65), ethyl-N-buthy-uralsaponin A esters (GU-66), gancaonin B (GU-67), methyl-24-hydroxyglycyrrhetate (GU-68), glyuranolide (GU-69), glyeurysaponin (GU-70), 5,6,7,8-tetrahydro-4-methylquinoline (GU-71), gancaonin E (GU-72), licocoumarone (GU-73), isoliquiritigenin (GU-74), methyl 2-hydroxy-3,4-dimethoxy benzoate (GU-75), neoliquiritin (GU-76), ganoderic acid A (GU-77), sigmoidin B (GU-78), uralsaponin A (GU-79), licoricesaponine K2 (GU-80), uralsaponin B (GU-81), umbelliferone (GU-82), gamma-sitosterol (GU-83), glycycoumarin (GU-84), liquiritin (GU-85), liquoric acid (GU-86), gmelofuran (GU-87), methyl-24-hydroxy-11-deoxoglycyrrhetate (GU-88), hispidulin (GU-89), isotrifoliol (GU-90), liquiritigenin (GU-91), glycyrrhisoflavanone (GU-92), 3-methyl-6,7,8-trihydropyrrolo[1,2-A]pyrimidin-2-one (GU-93), licoleafol (GU-94), tetrahydropalmatine (GU-95).

## Data Availability

The majority of the data used to support the findings of this study are included in the article. Other data are available from the corresponding author upon request.
